# Factors Affecting Client Satisfaction and Dissatisfaction in Out-Patient Rehabilitation Centers in Kurdistan Province in Iran

**Published:** 2012-02-01

**Authors:** N Hatamizadeh, P Jafary, R Vameghi, A Kazemnezhad

**Affiliations:** 1 Pediatric Neurorehabilitation Research Center, University of Social Welfare and Rehabilitation Sciences, Tehran, Iran; 2 Department of Rehabilitation Management, University of Social Welfare and Rehabilitation Sciences, Tehran, Iran; 3 Department of Pediatrics, Pediatric Neurorehabilitation Research Center, University of Social Welfare and Rehabilitation Sciences, Tehran, Iran; 4 Department of Biostatistics, Tarbiat Modares University of Medical Sciences, Tehran, Iran

**Keywords:** Client satisfaction, Rehabilitation, Public, Private

Dear Editor,

High quality health services need not only be universally accessible but also utilized by those for whom they are meant. Client satisfaction is an important determinant of health service utilization. According to Keegan, satisfaction comprises both cognitive and emotional facets and is related to previous experiences, expectations and social networks.[1] Fulfilling clients' satisfaction in rehabilitation services is important from different aspects. It is a pre-requisite for patient compliance and adherence to instructions.[2][3][4][5][6] Patients satisfied with their care are more likely to return for future care and recommend their healthcare provider to others.[4][7][8][9] In the present research, we studied the factors related to client satisfaction and dissatisfaction in rehabilitation services.

During a six months period, 415 patients were recruited from all of the public and private rehabilitation clinics existing in the Kurdistan Province of Iran using stratified sampling method. M/F ratio was 202/197. More than 50% of the participants were in the 25-44 year-old age group. A telephone interview was carried using a questionnaire which was a newly developed Iranian rehabilitation client satisfaction questionnaire and consisted of two main parts. The first part intended to measure and score clients’ satisfaction.[10] The second part which is the point of interest of this article was a semi-structured questionnaire for determining the factors perceived by clients as the most satisfying and the most dissatisfying, stated as clients’ “likes and dislikes”, by interview.

After completing the interviews, the responses were reviewed and coded by multiple researchers. Codes were grouped together under larger themes. Factors mentioned by clients as the major satisfying and major dissatisfying factors, were categorized into 7 main dimensions; physical environment, physical accessibility, time accessibility, financial accessibility, quality of care and equipments, personnel’s social interaction, and equity.

The five most frequently stated ‘negative dissatisfying situation’s were: lifelessness of the environment mentioned by both public and private center clients, no insurance coverage mentioned only by private center clients, closed afternoon hours mentioned only by public center clients, having stairs mentioned only by private center clients and narrow corridors for wheelchair passage mentioned by both public and private center clients.

The five most commonly stated ‘positive satisfying situations’ were therapist's precise answers to the clients' questions mentioned by both public and private center clients, reasonable fees and low costs mentioned only by public center clients, the admittance personnel's good, kind and calm behavior mentioned only by private center clients, the therapist's kind and empathic behavior mentioned by both public and private center clients, open afternoon hours mentioned only by private center clients and being under the coverage of all insurance companies mentioned only by the clients of public centers.

Surprisingly, to our knowledge, current literature on client satisfaction rarely focuses on factors comprising the different dimensions of satisfaction in details. This is true despite the fact that knowledge about the details of client satisfaction is the key to reform in every kind of service. We have intentionally avoided this pitfall in the present study. According to our findings, we suggest that making the following changes is required for assuring more satisfying conditions in rehabilitation centers:

(i) In terms of physical characteristics: Utilization of lively colors and provision of sufficient light for the environment, (ii) In terms of time accessibility: Introducing different kinds of rehabilitation services in each center, especially those frequently required simultaneously by rehabilitation clients and also providing afternoon open hours, (iii) In terms of financial accessibility: provision of insurance coverage, and (iv) In terms of personnel’s social interaction: Training the logistic staff, especially the admittance personnel, on personal interactions, along with ongoing monitoring and feedbacks.
